# The efficacy and safety of acupuncture and nicotine replacement therapy for smoking cessation: study protocol for a randomized controlled trial

**DOI:** 10.1186/s13063-022-06384-9

**Published:** 2022-06-06

**Authors:** Qingsheng Wang, Runjing Dai, Hailiang Zhang, Xiaomei Jiang, Xiaojing Ma, Xiangrong Zhang, Shisan Bao, Dong Ren, Jingchun Fan

**Affiliations:** 1 Clinical College of Chinese Medicine, Gansu University of Chinese Medicine, Lanzhou, Gansu 730000 People’s Republic of China; 2 Pneumology Department, Affiliated Hospital of Gansu University of Chinese Medicine, Lanzhou, Gansu 730000 People’s Republic of China; 3 School of Public Health, Laboratory and Simulation Training Center, Center for Evidence-Based Medicine, Gansu University of Chinese Medicine, Lanzhou, Gansu 730000 People’s Republic of China; 4 Psychosomatic and Sleep Medicine, Gansu Gem Flower Hospital, Lanzhou, Gansu 730060 People’s Republic of China; 5 Health Center of Hekou Town, Xigu District, Lanzhou, Gansu 730060 People’s Republic of China

**Keywords:** Acupuncture, Nicotine replacement therapy, Smoking cessation

## Abstract

**Introduction:**

Tobacco hazard is one of the most serious public health problems, accounting for up to 6 million deaths worldwide p.a. We aim to determine the efficacy and safety of acupuncture and/or nicotine replacement therapy on smoking cessation.

**Methods:**

We will recruit 96 participants who are willing to quit smoking by acupuncture and/or nicotine replacement therapy in Chengguan, Xigu and Heping Districts, Lanzhou city, for multicenter randomized, double-blind, double-dummy controlled clinical trial. Following obtained the informed consent forms, all eligible participants will be randomly divided into 4 groups: (1) acupuncture combined with nicotine patch, (2) acupuncture combined with sham nicotine patch, (3) sham acupuncture combined with nicotine patch, and (4) sham acupuncture combined with sham nicotine patch. These participants will be treated with different intervention modalities for 8 weeks and then will be followed-up for 8 weeks. The SPSS 26.0 software will be applied to analyze the clinical effects and adverse reactions of different intervention measures for smoking cessation.

**Discussion:**

This trial is a prospective, pragmatic, randomized, multicenter trial study protocol. The outcomes will illustrate the efficacy and safety of acupuncture and/or nicotine patches for smoking cessation. Provide smokers with a superior smoking cessation program.

**Trial registration:**

Chinese Clinical Trial Registry ChiCTR2100042912. Registered on January 31, 2021.

**Supplementary Information:**

The online version contains supplementary material available at 10.1186/s13063-022-06384-9.

## Introduction

Tobacco dependence is a chronic addictive disease, encoded as F17.21 in the International Classification of Diseases (ICD-10) [1]. Because cigarettes contain more than 70 carcinogens [2], long-term smokers can cause multi-organ and tissue damage, e.g., respiratory, cardiovascular, and neuromuscular diseases, and even death [3]. Quitting smoking is one of the most effective approaches to improve health condition and save lives. The benefit of reducing/quitting smoke is evidenced in improving the quality of life via decreasing the mortality and morbidity of cancer and cardiovascular diseases [4, 5]. Although there are 40% smokers who are/were willing to quit, only 8% are quitting successfully for multiple reasons [6]. Therefore, it becomes critical important to explore the effective approach to assist these remind unsuccessful quitting smokers to achieve their goal.

Nicotine replacement therapy (NRT), considered the basic treatment for smoking cessation, is the main pharmacological smoking cessation modality recommended by WHO [7]. Currently, NRT includes nicotine patches, nicotine gum, nicotine nasal sprays, and nicotine inhalers. Although nicotine patches are available for a long time within the most effective in smoking quitting [8, 9], the safety of nicotine patches is debated, partially is due to its adverse reactions, e.g., dizziness, headache, and nausea [10]. Acupuncture is a relatively common approach for smoking cessation over the past decades [11] with benefit of short-term abstinence, safety, and economic costs. During smoking cessation acupuncture significantly improves withdrawal symptoms, including upset, irritability, anxiety, and impatience experienced. However, the precise role of acupuncture for smoking cessation requires further clarification [12, 13]. Hyun S et al. demonstrate that NRT and behavioral counseling combined with auricular acupuncture are safer and more effective than traditional therapy alone [14]. However, it is still controversial about the efficacy of smoking cessation in acupuncture, NRT, and other interventions in clinical trials or related meta-analysis studies [15, 16], which remains to be validated.

It is reported that combined approach is more effective than a single intervention in improving the successful rate of smoking cessation. Therefore, this trial will use a double-blind and double-dummy randomized controlled trial, to explore the efficacy and safety of acupuncture and/or nicotine patch with placebo on smoking cessation, so as to enrich and optimize smoking cessation programs and provide effective smoking cessation methods with lower relapse rates for smokers.

## Methods

### Objectives

The objective is to explore the effect and safety of different interventional approaches, e.g., acupuncture with or without nicotine patch, nicotine patch, and placebo for smoking cessation.

### Study design

**Fig. 1:**
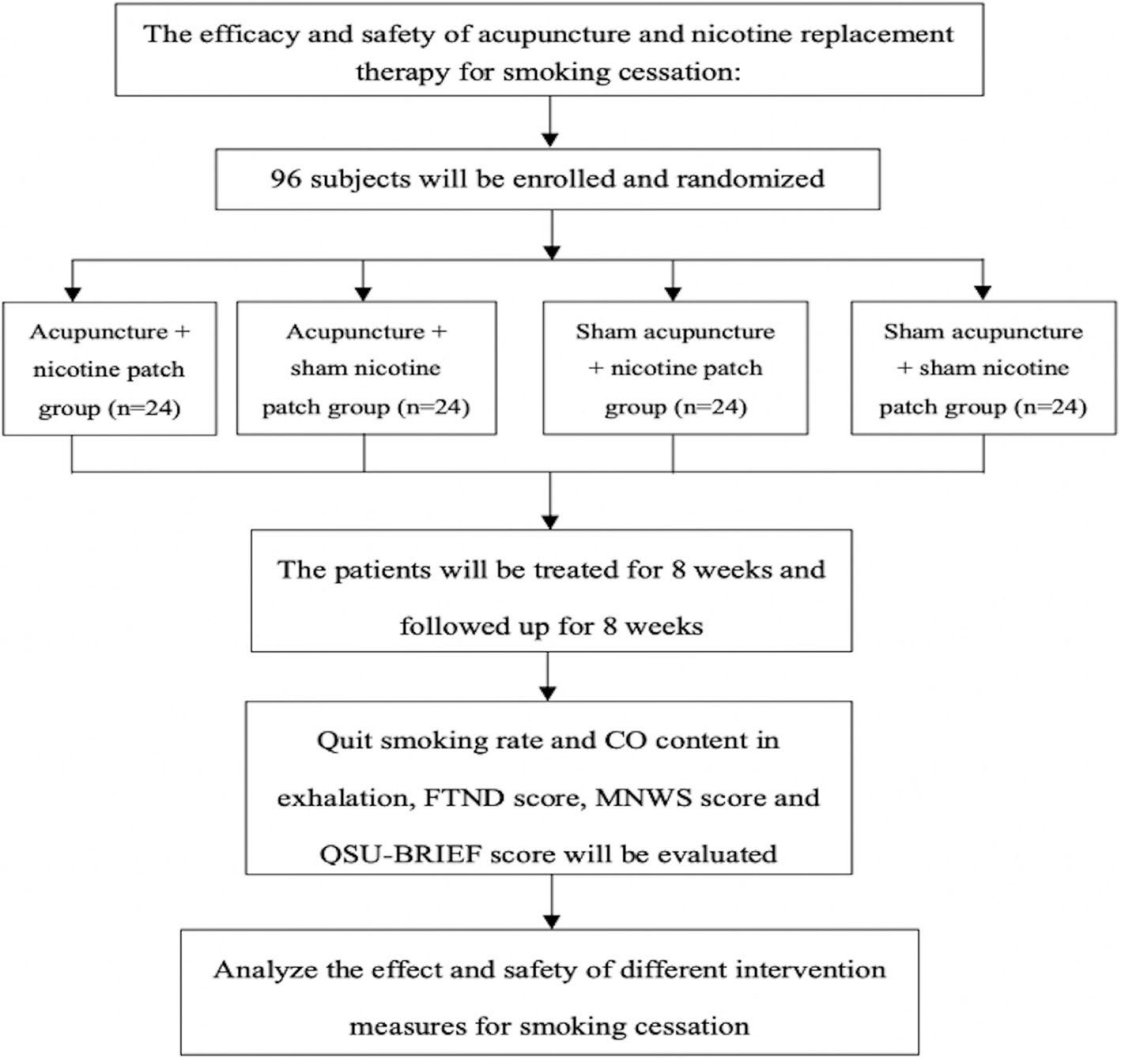
The flowchart of research scheme

This study will be used a randomized, controlled, double-blind, double-dummy, multiple-center experimental design. We will follow the ethical principles of the Declaration of Helsinki and adhere to the principles of good clinical practice. The study is registered in the Chinese Clinical Trial Registry (registration number: ChiCTR2100042912) on January 31, 2021. The diagram is illustrated that the different phases of the study (Fig. 1 and Table 1). The Standard Protocol Items: Recommendations for Intervention Trials (SPIRIT) checklist is provided as Additional file 1 [17].

**Table 1:**
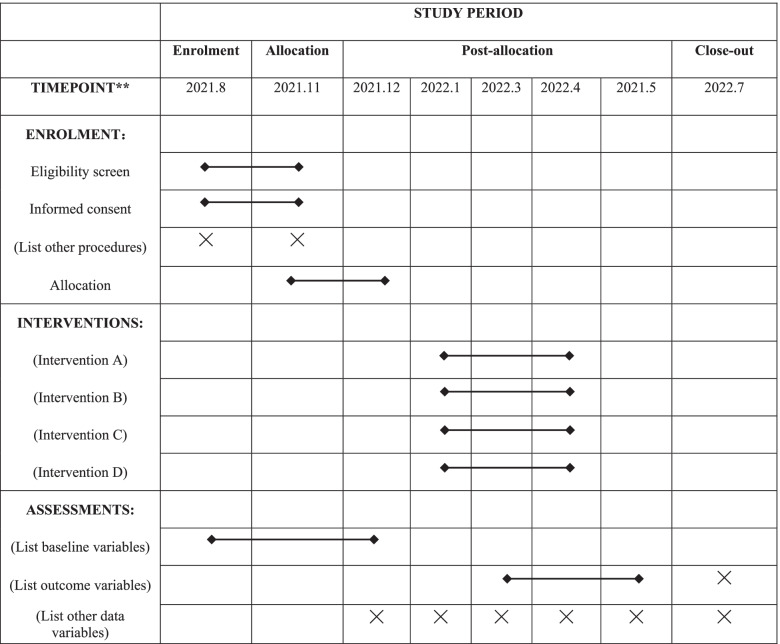
The schedule of enrolment, interventions, and assessments

### Eligibility criteria of the participants

#### Diagnostic criteria

According to the Diagnostic and Statistical Manual of Mental Disorders (DSM-IV) prepared by the American Psychiatric Association, Diagnostic Criteria for Nicotine Dependence 292.0:

1. Daily use of nicotine for at least 3 weeks
2. More than 4 of the symptoms that occur within 24 h after stopping or reducing smoking: dysthymia or depression, insomnia, irritability, anger, anxiety, difficulty concentrating, restlessness, slow heart rate, and increased appetite or weight gain
3. Clinically significant distress due to the above symptoms or functional deficits in social, occupational, and other important aspects
4. These symptoms are not due to a physical condition and cannot be attributed to any other mental disorder

#### Inclusion criteria

1. Meets diagnostic criteria for smoking
2. Smokers who voluntarily quitted smoking
3. ≥ 16 years
4. Informed consent

#### Exclusion criteria

1. Participants with severe heart, brain, lung related system diseases and diabetes
2. Participants who have received other relevant smoking cessation treatment within half a month
3. Allergic to nicotine patch or acupuncture

#### Dropout criteria

1. Occurrence of serious adverse events
2. The participants withdraw spontaneously
3. Participants develop serious diseases during treatment

### Sample size calculation

We will use the calculation formula of sample size of enumeration data in a completely random design, as describe previously. The PASS software will be used for calculation. The formula is listed as follow:

$$N=\frac{\Big[{Z}_{\alpha}\sqrt{2p\left(1-p\right)}+{Z}_{\beta }{\sqrt{p_1\left(1-{p}_1\right)+{p}_2\left(1-{p}_2\right)\Big]}}^2}{{\left({p}_1-{p}_2\right)}^2}$$

*α* = 0.05 will be set up as the level of test, and the test power is 90%. The withdrawal rates of acupuncture (*p*_1_) and placebo (*p*_2_) will be 56% [18] and 9.1% [19], respectively; *p* is the mean of *p*_1_ and *p*_2_. The PASS 26.0 software will be used to calculate that the sample size required for each group for ≥18 cases. Considering 30% loss to follow-up rate, the sample size per group will be 24 cases. Consequently, the total number of the participants will be 96, based on 24 case per group in the four groups.

### Randomization

A total of 96 eligible participants will be recruited from Chengguan, Xigu and Heping Districts, Lanzhou City, Gansu Province, by posters and the most popular and ubiquitous Chinese form of social media, WeChat.

1. Sequence generation: A series of consecutive numbers (from 1 to 96) will be generated prior to the recruitment, and computer software (Microsoft Excel 2016) will be used to randomly assign each number to one of the four intervention groups (1: acupuncture combined with nicotine patch group (ACNP), 2: acupuncture combined with sham nicotine patch group (ACSNP), 3: sham acupuncture combined with nicotine patch group (SACNP), and 4: sham acupuncture combined with sham nicotine patch group (SACSNP)
2. Allocation concealment mechanism: All will be performed in the double blind fashion. This randomization sequence will be inserted into sequentially numbered, opaque, sealed envelopes (to ensure allocation is concealed from assessors). These envelopes will be opened by the acupuncturist treating the patient

### Recruitment

We will recruit participants from three hospitals separately, involving in the smoking cessation based on the distribution of participants. Such recruitment will be broadcasted using a few approaches, including posters and the most popular and ubiquitous Chinese form of social media, WeChat, to establish contacts with various ethnic community, university, and business groups, and conducted community-based smoking cessation programs.

### Blinding

**Fig. 2:**
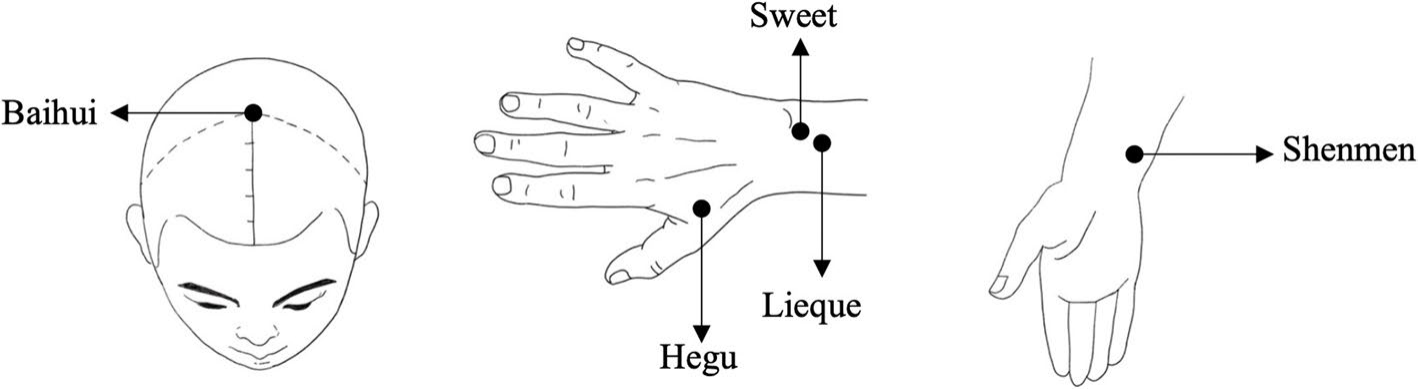
Acupuncture points map

**Fig. 3:**
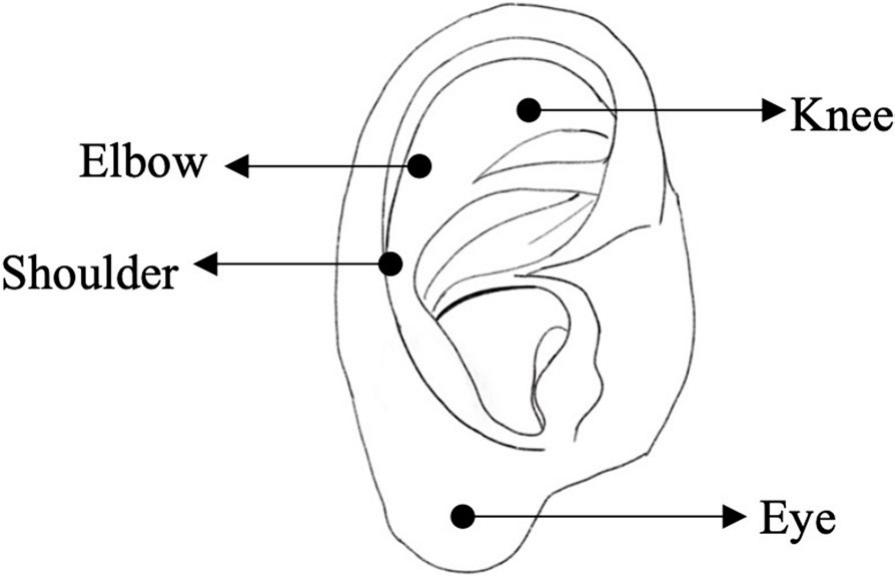
Sham acupuncture points map

In this study, a randomized, controlled, double-blind, double-dummy, multi-center experimental design method will be adopted. In view of the particularity of acupuncture manipulation, it is difficult to blind the practitioners of the study. Thus, we can only blind the participants and the data collectors. The specific operation process is as follows: prior to the study, the participants will sign the informed consent, and each participant will be informed that both acupuncture and patch treatment methods would be adopted. The research group will select two groups of acupuncture points (Fig. 2, Fig. 3), so the locations of each acupuncture point may be different, but we will not inform the specific names of the acupuncture points and their effects. Because placebo patches are the exact size and shape of the patch that has already been purchased, the participants will be masked the intervention received all the time. In addition, in our data collection process, specialized data collectors send questionnaires to the research objects in the form of “Questionnaire Star,” and the data collection is completed through the network. To ensure the correct application of blindness in this study, these participants will be not aware of the grouping of the research objects.

### Assignment of interventions (for controlled trials) allocation

#### Acupuncture or sham acupuncture

Acupuncture points Baihui, Lieque, Hegu, Shenmen, and Sweet points (Fig. 2) [12]. Participants will be placed in a supine position with adequate exposure of the selected acupoints and disinfection with 75% medical alcohol. For Hegu acupoint, 0.25 × 40 mm acupuncture needles will be inserted downward and vertically, and a disposable 0.25 mm–25 mm acupuncture needle will be used to insert the needle 0.5 inch downward at a 15° angle to the skin at the other acupoints. All acupoints will be fixed after needles insertion, and the presence of soreness and swelling sensation in the acupoints will be used as the standard.

Acupoints on the auricle corresponding to shoulder, eye, knee, or elbow will be taken with sham acupuncture (Fig. 3) [20]. Except for the eye points, acupuncture needles of 0.18 mm × 13 mm will be used to prick downward at a 15° angle with the skin, and the eye points will be inserted vertically into the needles and fixed.

The needle will be kept for 20 min for each time after insertion. During the period of needle retention, group 1 and group 2 will be given needle every 10 min, and a dry cotton ball will be used to press the pinhole when the needle is released. The participants will be acupunctured three times a week from week 1 to 4, and twice a week from week 5 to 8. Thus, total acupuncture time will be 8 weeks.

#### Nicotine or sham nicotine patches

The nicotine or sham patch will be stuck on the chest, upper arm, wrist, or thigh of each participant, ensuring the patches are kept dry and clean. The specific course and dosage are shown (Table 2).

**Table 2:** Duration and dosage of nicotine patch

Daily smoking/weight	Week 1-2	Weeks 3–4	Weeks 5–8
≥ 20	2 patches/day	1 patch/day	0.5 patches/day
< 20/≥70 kg	2 patches/day		
< 20/< 70 kg	1 patch/day		

### Baseline data

We will collect basic information including age, gender, ethnicity, educational level, BMI, blood pressure, heart rate, marital history, smoking history, daily smoking amount, times of quitting, smoking status of family members, and friends in the questionnaire.

### Outcomes

#### Primary outcome

Abstinence rate, relapse rate, and expiratory CO value will be selected as the main outcome indexes. The former two are mainly judged by the self-report of the participants at the end of treatment and follow-up, while the expiratory CO content is mainly detected by the CO detector.

#### Secondary outcome

Secondary measures included a modified Fagerstrom test for nicotine dependence (FTND) score, a Minnesota Nicotine Withdrawal Scale (MNWS), and modified Brief Questionnaire of Smoking Urge (QSU-Brief) score. The three scores will be used to reflect the severity of nicotine dependence, withdrawal symptoms, and smoking craving of the participants. The higher scores indicate, the more severe symptoms. Each patient will be asked a questionnaire prior to the treatment and at 1, 4, and 8 weeks after the treatment. Table 3 shows the specific observation points of each index during the study.

**Table 3:** The measurement time points of various indicators

Indicators	Before start of treatment	Treatment period			End of the follow-up
		Week 1	Week 4	Week 8	
Abstinence rate				√	√
Relapse rate					√
Expiratory CO value	√			√	
FTND scores	√	√	√	√	
MNWS	√	√	√	√	
QSU-Brief scores	√	√	√	√	

### Adverse events

Adverse events in this study included needle fainting, stagnation, bending, folding, and allergy during the use of the patch, as well as adverse reactions such as bleeding, hematoma, dizziness, fainting, residual needle sensation, tenderness, and infection during the treatment. In case of any adverse reaction, the physician will take protective measures for the subject in time and decide whether to stop the trial based on the disease condition and experience director. The study director will record the time, degree, duration, and treatment measures of the occurrence on the observation sheet and indicate the date of the report, to facilitate the later evaluation of the correlation between the occurrence and the test.

### Data management and monitoring

We will collect the data by issuing questionnaires through WeChat in the form of “Questionnaire Star.” To enhance the accuracy of the data and maintain the quality of the data. All the data collected will be checked and collated by two dedicated data managers and then import into the Excel. These data will be kept confidentially until the end of the trial and will not be available to anyone except for the data administrator.

Our study will start in August 2021, and the specific study stages and procedures are shown in Fig. 1 and Table 1.

### Statistical analysis

The SPSS 26.0 software will be used for analysis. Measurement data will be expressed as *mean ± SD*, and enumeration data will be expressed as rate. Repeated measurement analysis of variance will be used for intra-group comparison of measurement data satisfying normal distribution, and SNK (Student-Newman-Keuls) method will be used for inter-group comparison; quantitative data will be tested by *χ*^*2*^. Nonparametric tests will be used for data that do not meet the normal distribution and equal variances. *P* ≤ 0.05 will be considered significant. PPS (per-protocol set) will be used in the current study, and only the data will be analyzed from the participants who will perform the whole process. For those who lack follow-up data, the data of the last observation carried forward.

### Participant’s management system

The informed consent will be obtained to all of the participants prior to the conduct of the study. The testers will be followed up regularly and given verbal spiritual encouragement to minimize dropout.

### Ancillary and post-trial care

At the end of the trial, we will provide free nicotine patch to the participants who have not achieved the cessation goal or effect until the desired effect is achieved.

## Discussion

Smoking is not only a physical dependence, but also a psychological dependence, thus, smoking cessation requires more than a strong will [21]. This study can only be carried out when the participants have certain intentions to quit smoking. We will choose two methods of NRT and acupuncture for the combined intervention of smokers who are willing to quit. Acupuncture is a relatively common and effective method in traditional Chinese medicine, while NRT is the most commonly used of the internationally recognized interventions. This combination is rarely reported in the literature, and it is expected to achieve better outcomes. We know that smoking behavior falls under the category of “mental and behavioral disorders resulting from the use of psychoactive substances” [22]. Thus, the choice of control group will not be a single intervention, but a combination of a placebo treatment of another method, as well as a group of pure placebo group. Each group will receive two interventions designed to blind the participants and provide a psychological cue that will help improve the effect of the intervention and promote participant compliance with the regulations.

There is a paradigm shift towards patient-centered care and active involvement of participants and public in research design and active participation throughout the research process [23]. However, to ensure the implementation of blind method in the later stage of the experiment, this paradigm has not adopted in the design of this study, and the protocol designers will be all composed of trial researchers, without the participation of the public or participants.

There are several limitations in the current study. First, although we chose the double dummy to try to blind the study participants, we are unable to blind the physicians, which may lead to some bias. However, all the subjective indicators are collected through the network background, which will minimize these biases to some extent. Secondly, although we have performed the sample size calculation, the sample size is still small, and a large sample test will be needed in the future stage.

Our study will evaluate the efficacy and safety of different interventions for smoking cessation, providing some evidence for smokers to choose smoking cessation programs, as well as for future studies, and promote the development of acupuncture for smoking cessation. The results will be published within 6 months after the trial completed.

### Trial status

The study registration number is ChicTR2100042912, and the recruitment is still ongoing. Recruitment may end in October 2020.

## Supplementary Information

**Supplementary file 1.** SPIRIT Checklist for Trials.

## Abbreviations

ACNP: Acupuncture combined with nicotine patch
ACSNP: Acupuncture combined with sham nicotine patch
SACNP: Sham acupuncture combined with nicotine patch
SACSNP: Sham acupuncture combined with sham nicotine patch
FTND: Fagerstrom test for nicotine dependence
MNWS: Minnesota Nicotine Withdrawal Scale
QSU-Brief: The Brief Questionnaire of Smoking Urge
SNK: Student-Newman-Keuls
